# Perceived effectiveness of learning methods among preclinical medical students - role of personality and changes over time

**DOI:** 10.12669/pjms.37.7.4355

**Published:** 2021

**Authors:** Shoukat Ali Arain, Daeya Ahmad Alhadid, Shahzad Rasheed, Maram Mansour Alrefaai, Tarek M. Ahyaf Alsibai, Sultan Ayoub Meo

**Affiliations:** 1 Shoukat Ali Arain, MBBS PhD. Department of Pathology, College of Medicine, Alfaisal University, Riyadh, Saudi Arabia; 2 Daeya Ahmad Alhadid, MBBS. College of Medicine, Alfaisal University, Riyadh, Saudi Arabia; 3 Shahzad Rasheed, MBBS M. Phil. Department of Anatomy, College of Medicine, Al-Imam Mohammad Ibn Saud Islamic University, Riyadh, Saudi Arabia; 4 Maram Mansour Alrefaai, MBBS. College of Medicine, Alfaisal University, Riyadh, Saudi Arabia; 5 Tarek M. Ahyaf Alsibai, MBBS. College of Medicine, Alfaisal University, Riyadh, Saudi Arabia; 6 Sultan Ayoub Meo, MBBS PhD. Department of Physiology, College of Medicine, King Saud University, Riyadh, Saudi Arabia

**Keywords:** Personality, Active learning, Academic performance, Undergraduate medical education, Problem-based learning

## Abstract

**Background and Objectives:**

Active learning methods are vital in inculcating skills of critical thinking, lifelong learning and effective communication. Personality may influence learning method preferences and academic performance. The aim of this cross sectional study was to examine the relationship between students’ personality and their predilection for learning methods and academic performance.

**Methods:**

Perceived effectiveness of learning methods was assessed over time. Second- and third-year medical students (n=112) completed a questionnaire consisting of Big Five Inventory to measure the personality dimensions, and evaluated lecture, problem-based learning (PBL) and team-based learning (TBL) for their helpfulness in learning. Grade point average (GPA), PBL and TBL grades were obtained. Correlation coefficients were calculated between personality traits and learning method effectiveness scores, and grades. Learning methods effectiveness was compared between second- and third-year students.

**Results:**

Positive correlations were identified between conscientiousness and lecture (r = 0.30), agreeableness and lecture (r = 0.20), and agreeableness and TBL (r = 0.23). Likewise, positive correlations were seen between extraversion and PBL grade (r=0.20), and conscientiousness and GPA (r = 0.23). In third year, significant decline in perceived effectiveness of lecture was seen (81% vs 57%; p = 0.006), while increased perceived effectiveness for PBL (38% vs. 50%) was not statistically significant (p = 0.22).

**Conclusions:**

The findings provide an evidence for modest correlations between personality and perceived effectiveness of learning methods. Remarkably, perceived effectiveness decreased for the lecture and increased for the PBL over time. The findings may help educators in better implementing active learning modalities. Besides, an earlier introduction may help students becoming acquainted with and getting the most out of PBL.

## INTRODUCTION

To prepare medical students as competent healthcare providers, medical education must be dynamic to be able to adapt to the new knowledge and the changing nature of the work environment.^1^ There is growing need for improved care coordination and effective communication between multidisciplinary teams in complex healthcare settings.^2^ Consequently, educational models emphasizing critical thinking, lifelong learning skills, and effective communication and teamwork, in the form of collaborative and active learning, are being incorporated into medical curricula.^3^,^4^

Problem-based learning (PBL) and team-based learning (TBL) are prime examples of such ‘student-centered’ and collaborative active learning strategies. In PBL, real-life clinical scenarios are introduced to students in small groups. Specific ‘triggers’ in these scenarios provide the context for brainstorming and learning. Students analyze, inquire, explore and exchange information based on the data given in the scenario. The whole process is facilitated and supervised by a trained faculty member who also grades students’ individual performance^5^. Similarly, TBL is an expert-led interactive and collaborative learning method. Before each session, students are provided with a set of learning objectives along with learning material. During the session, students take readiness assurance test (RAT), consisting of 10-15 multiple choice questions, first individually (iRAT) and then in teams (tRAT). Besides, students apply the gained knowledge, as teams, in solving clinical scenarios.^6^ For the successful implementation of such methods, considering student variability and preferences is important. Increasingly, the role of personality traits in predicting students’ preferences for learning methods and academic performance has been examined.^7^

Personality traits can be narrowed down into five categories; extraversion (outgoing individuals who tend to be full of energy), conscientiousness (organized, ambitious and detail-driven individuals), agreeableness (kind, sympathetic and cooperative individuals), openness to experience (adventurous, creative, and curious individuals) and neuroticism (emotionally unstable and vulnerable to stress and anxiety).^8^,^9^ These traits are predictive of a wide range of behaviors and important life outcomes including academic and career achievements.^10^

Personality dimensions of medical students are known to significantly influence learning styles.^11^,^12^ However, previous studies have mostly been centered on preferred learning styles, rather than specific learning methods. In few studies, described below, preferences for interactive and non-interactive learning methods have been reported. Interactive teaching positively correlated with a combination of emotional stability and agreeableness, while extraversion was negatively associated with the independent study^13^,^14^ reported that extraversion positively correlated to the “assist-coordinate” characteristic, while conscientiousness was positively related to the “control-lead” characteristic. Likewise, Holen *et al*. (2015)^15^ reported a significant positive correlation of all personality traits with PBL except neuroticism.

Furthermore, a number of studies have correlated personality traits with GPA and clinical performance^16^,^17^ while data are scarce on medical students’ academic performance in active learning methods in preclinical years. Hence, grades in the educational environment of active learning methods also need to be examined in the context of personality dimensions as a reflection of their effectiveness in learning.

Based on previous reports, we hypothesized a role of personality traits in students’ preference for, and academic performance in active learning methods; PBL and TBL. Therefore, this study aimed to examine the relationship between medical students’ personality traits, their self-perceived effectiveness of learning strategies, and academic performance. Also, the perception of second- and third-year medical students was compared to evaluate any changes in the perceived effectiveness of various learning strategies at different stages of the curriculum.

## METHODS

### Institutional context

At College of Medicine, Alfaisal University Riyadh, Saudi Arabia, a five-year, organ-system based MBBS curriculum is completed in 10 semesters. It is designed in spiral fashion, emphasizing a gradual “basic to clinical” shift in themes and training. Both active and passive (hybrid) teaching learning strategies are in place. Problem-based learning (PBL) and team-based learning (TBL) remain major active learning strategies during the first three years.

A 44-item questionnaire, the Big Five ***Survey design:*** In this cross-sectional study, second- and third-year students were invited to complete an electronic survey. All the students who filled out the survey, and had their grade point average (GPA), PBL and TBL grades available, were included in the study. The study was approved by the Institutional Review Board (Ref # IRB-040-17, Dated: 04/05/2017).

Inventory (BFI) was used in this study to measure personality dimensions, which is shorter and easier to comprehend compared to other similar tests and thus easier to administer in large groups^18^. Besides, it shared similar psychometric measurement properties to other extended versions with a satisfactory internal consistency.^19^,^10^ Participants were also asked to evaluate lecture, PBL and TBL sessions for their helpfulness in learning on a five-point Likert scale. PBL and TBL grades and GPA were also obtained.

As distinct modules are offered in second and third year, to keep the data comparable second year grades were used for all participants (regardless of their current year of study). PBL grades were given to individual students by PBL facilitators based on a structured grading rubric incorporating interpersonal skills, participation, and knowledge. TBL grades were assigned based on individual readiness assurance test (iRAT), team readiness assurance test (tRAT) and peer evaluation scores.

### Data analysis

Data were entered in Microsoft excel and transferred to the IBM SPSS Statistics for Windows, Version 22.0 (Armonk, NY: IBM Corp.) for statistical analysis. The internal consistency of BFI was evaluated through the calculation of Cronbach’s alpha. Initially, with 44-item BFI, Cronbach’s alpha (α) value was found to be 0.70 with a value of 0.64 for the items of openness to experience. Item number 41, “Has few artistic interests” had a corrected item-total correlation of -0.185. Removing this item improved the α value for the openness items to 0.73 and for 43-item BFI to 0.71. Although, α value for the items of agreeableness was also low (.65), the removal of any item did not improve it significantly. Thus, 43-item BFI was used for further analysis. Table-I depicts the internal consistency for each subscale of BFI.

**Table I:** Internal consistency of subclasses in 43-item BFI.

*Personality trait*	*Items *N**	*Cronbach’s alpha*
Agreeableness	9	0.65
Conscientiousness	9	0.75
Extraversion	8	0.80
Neuroticism	8	0.79
Openness	9	0.73

BFI: Big Five Inventory.

Means scores were calculated for personality traits and preferred learning methods based on Likert-scale data. Mean scores for perceived effectiveness of learning methods were compared using One-way ANOVA. Correlation coefficients were calculated between personality-trait mean scores, and students’ perceived effectiveness for learning methods and grades using Spearman’s rho for categorical and Pearson correlations for continuous variables. A correlation coefficient (r) value of ≥ 0.20 was considered weak, ≥ 0.30 as moderate and ≥ 0.40 as strong correlation.^20^ Besides, Likert scale data for perceived effectiveness of learning methods was also calculated as percent agreement by combining strongly agree and agree. These percent agreement scores were compared between 2^nd^ and 3^rd^ year students using chi-square test. A p-value of < 0.05 was considered significant.

## RESULTS

Responses from 112 students were included in the final analysis, of which 87 (77.7%) were female students and 60 (53.6%) were from Year-3. A summary of the questionnaire and performance data are presented in Table-II. The mean personality trait score was highest for the openness trait [3.60 (SD 0.47)], and overall, lecture was perceived as the most effective learning method (p <0.001).

**Table II:** Summary of the questionnaire and academic performance data.

***Gender***	***n (%)***
Male	25 (22.3)
Female	87 (77.7)
***Academic level***	***n (%)***
Year 2	52 (46.4)
Year 3	60 (53.6)
***Personality traits score***	***mean (SD)***
Agreeableness	3.20 (0.21)
Conscientiousness	3.33 (0.21)
Extraversion	3.08 (0.23)
Neuroticism	3.10 (0.23)
Openness	3.60 (0.47)
Preferred learning method*	***mean (SD)***
Lecture	3.85 (1.10)
PBL	3.15 (1.22)
TBL	3.21 (1.33)
***Grades***	***mean (SD)***
PBL (out of 15)	13.40 (0.95)
TBL (out of 10)	8.03 (0.71)
GPA (out of 4)	3.46 (0.39)

* p-value <0.001 using One-way ANOVA.

Correlation coefficient values between personality-trait mean scores, and students’ perceived effectiveness of the learning strategies and grades are shown in Table-III. Agreeableness was positively correlated with traditional lecture and TBL. Conscientiousness showed a positive correlation with the lecture and GPA. Extraversion showed a positive correlation for PBL grade. Correlation ranged from weak to moderate. Neuroticism and openness to experience did not show any correlation either for learning methods or grades.

**Table III:** Correlation coefficients between personality-trait mean scores, and students’ perceived effectiveness scores for learning methods and grades (n = 112).

	*Spearman’s rho*			*Pearson Correlation*		

*Personality Trait*	*Preference score*			*Grade*		*GPA*

	*Lecture*	*PBL*	*TBL*	*PBL*	*TBL*	
Agreeableness	0.20*	0.07	0.23*	-0.14	0.15	0.03
Conscientiousness	0.30†	0.07	0.16	-0.04	0.14	0.23*
Extraversion	0.06	0.18	0.15	0.20*	0.10	0.06
Neuroticism	0.13	0.11	0.00	0.08	0.10	0.17
Openness	0.07	0.01	-0.02	0.00	0.09	0.18

* weak, †moderate correlation. PBL; Problem-based learning, TBL; Team-based learning, GPA; Grade point average.

**Fig.1:**
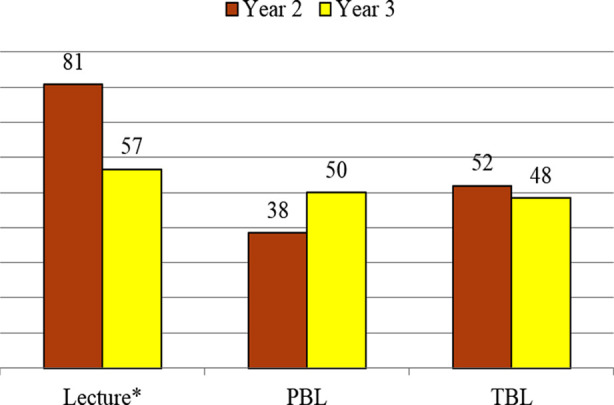
Year-wise perceived effectiveness for learning strategies. Results are shown as percent agreement. PBL; Problem-based learning, TBL: Team-based learning. *P-value is significant using χ² test (p = 0.008).

Comparison of perceived effectiveness for the learning methods between second- and third-year students (Fig.1) showed a significant decline for lecture in third year (81% vs 57%; p = 0.006). Conversely, perceived effectiveness for PBL increased from 38% to 50%, though the increase was not statistically significant (p = 0.22). However, preference for TBL remained relatively constant for second- and third-year students (52% vs. 48%).

## DISCUSSION

The present study reports certain pertinent correlations between personality traits, perceived effectiveness of learning methods and academic performance. In personality subscales, modest correlations were found between higher agreeableness score and a preference for the traditional lecture (r = 0.20) and TBL (r = 0.23), higher conscientiousness score for a better GPA (r = 0.23) and a preference for the lecture (r = 0.30), and higher extraversion scores for a better PBL grade (r = 0.20). Intriguingly, in third year, perceived effectiveness decreased for lecture (81% vs 57%; p = 0.006) while it did not increase significantly for PBL (38% to 50%; p = 0.22).

The reliability of the BFI has been found satisfactory in previous studies with Cronbach’s alpha values for all the subscales usually ranging from close to or above 0.80^19^. In the present study, after the removal of one item for openness, overall Cronbach’s alpha for 43-item BFI remained at an acceptable value of 0.71. It was slightly low (0.65) for the subscale of openness 21. A possible reason for the relatively low Cronbach’s alpha could be the variable comprehension of the phrasal language used in the inventory. Although English is the medium of instruction, most students were non-native English speakers from a wide range of lingual and cultural backgrounds.^22^

Students with high agreeableness preferred both lecture and TBL. Individuals high in agreeableness get along with others and behave well in interpersonal interactions. However, such individuals are sensitive and tend to avoid conflict and confrontation. Perhaps such avoidance of conflict drove preference for lecture compared to PBL in this group.^15^ Similarly, compared to PBL, TBL has lesser component of group discussion and conflict, an environment in which agreeable students are expected to get along well. Additionally, having lesser exposure to the PBL can be a contributing factor. In fact, agreeableness score was significantly higher in students of second year (p = 0.03) who were involved in the PBL only for less than six months.

Students with higher conscientiousness are known to be organized, persistent and hardworking, and can regulate their impulses.^23^ They preferred lecture possibly due to them being considered a more efficient and organized vehicle of knowledge delivery. Indeed, an association has been reported between conscientiousness and obsession for structure and order.^24^ Conversely, essential components of collaborative active learning methods, especially of PBL, are disorganized brainstorming and a ‘messier’ acquisition of knowledge which can be considered disarray and chaos by the organized and careful conscientious students.^15^

Keeping with their personality, those high in extraversion had higher grades in PBL. Students were graded based on their participation, in terms of analyzing the problem, presenting their thoughts and interpersonal skills. Our findings are consistent with earlier studies showing that extravert medical students generally perform better in groups with significant interpersonal interactions^21^. Besides, it is known that those high in extraversion prefer PBL.^11^ In fact, our cohort demonstrated that very tendency (r = 0.18).

Collaborative, active learning methods are important in developing interpersonal and social skills deemed advantageous for physicians. However, our cohort showed a significant preference for traditional lecture. Possibilities include cultural background, a high-school entry taught in the teacher-centered and exam-focused curriculum, variable language fluency as most students are non-native English speakers and lack of training in collaborative active learning methods.^25^ An important confounding factor in our cohort could also have been the duration of exposure to each learning method. Lecture and TBL were introduced from year one, while PBL was introduced at the beginning of year two. Thus, second year students were involved in the complex process of learning through PBL for only less than six months.

Remarkably, in third year, the percent agreement for perceived effectiveness of lecture declined significantly while it improved for the effectiveness of PBL. Although, third year students were a different cohort, we believe that their better perception about the effectiveness of PBL was due to their extended experience in the strategy as supported by the earlier reports.^25^

Our findings have important implications. For instance, academic performance of extraverts improves as academic activities involving interpersonal interactions increase in the curriculum during advanced years.^26^,^12^ Besides, they are known to prefer PBL^5^. In our cohort, extraverts performed better in PBL, suggesting that the academic performance of extraverts can be improved even in preclinical years through engaging them in collaborative, active learning methods. Similarly, those high in agreeableness also preferred TBL in addition to lecture. The complex learning process of PBL might have been hectic for them as they value getting along with others. They may benefit from the inclusion of TBL or a better structured PBL.^27^

Moreover, students who perceive lecture as being more effective might have difficulties in adjusting to the new ways of collaborative learning signifying the need for appropriate guidance in adapting to new environments. Importantly, perceived effectiveness of PBL increased over time as students learnt the art of acquiring knowledge through complex collaborative learning. Inclusion of collaborative learning methods early in the curriculum are likely to maximize their value.

### Study Limitations

This is a cross-sectional survey with the participation of a limited number of preclinical students under specific conditions from a single institute. Findings may not necessarily be generalized. Generally, preclinical curricula are heavily overloaded with factual knowledge and students tend to be more competitive and impersonal. It would be intriguing to explore the causal relations involving a larger number of participants, that can help faculty and curriculum managers to device strategies to customize these methods for struggling students. Besides, attitudes towards these methods may change with extended experience and with a realization for the need of interpersonal skills and teamwork. Hence, interesting would be the exploration of changes in this relationship over time with inclusion of collaborative learning method, especially PBL, earlier in the curriculum.

## CONCLUSIONS

Our findings provide evidence for modest correlations between personality traits and students’ perceived effectiveness of learning methods. Those students high in agreeableness perceived lecture and TBL as most effective while conscientious students showed a predilection for lecture and had a better GPA. Extraverts achieved a better grade in PBL. However, neuroticism and openness to experience neither showed a predilection for any learning method nor correlated to academic performance. Compared to second year, students of third year regarded lecture less effective and PBL more effective in acquiring knowledge. These findings may help educators to better understand and provide appropriate support to the students at risk of struggling in collaborative learning methods. Besides, an introduction of the PBL earlier in the curriculum may help students becoming acquainted with and gaining most out of this collaborative learning method.

### Author Contributions:

**SAA, DAA, MMA:** Conceived and designed research, drafted manuscript, are responsible for integrity of research.

**SR:** Analyzed data.

**TMAA, SAM:** Edited and revised manuscript; all authors approved final version of manuscript.
